# Screening of an individualized treatment strategy for an advanced gallbladder cancer using patient-derived tumor xenograft and organoid models

**DOI:** 10.3389/fonc.2022.1043479

**Published:** 2022-12-14

**Authors:** Dengxu Tan, Jiaze An, Miaomiao Gong, Huihui Wang, Han Li, Han Meng, Caiqin Zhang, Yong Zhao, Xu Ge, Changhong Shi

**Affiliations:** ^1^ Division of Cancer Biology, Laboratory Animal Center, Fourth Military Medical University, Xi’an, Shaanxi, China; ^2^ Gansu Provincial Laboratory Animal Industry Technology Center, Gansu University of Chinese medicine, Lanzhou, Gansu, China; ^3^ Department of Hepatobiliary and Pancreaticosplenic Surgery, Xijing Hospital, The Fourth Military Medical University, Xi’an, China; ^4^ School of Basic Medical Sciences, Medical College of Yan’an University, Yan’an, China; ^5^ Group 4, School of Basic Medicine, Air Force Military Medical University, Xi’an, Shaanxi, China

**Keywords:** gallbladder cancer, patient-derived tumor xenograft model, patient-derived tumor organoid model, individualized therapy, immune checkpoint inhibitors

## Abstract

Gallbladder cancer is a highly aggressive malignancy with poor sensitivity to postoperative radiotherapy or chemotherapy; therefore, the development of individualized treatment strategies is paramount to improve patient outcomes. Both patient-derived tumor xenograft (PDX) and patient-derived tumor organoid (PDO) models derived from surgical specimens can better preserve the biological characteristics and heterogeneity of individual original tumors, display a unique advantage for individualized therapy and predicting clinical outcomes. In this study, PDX and PDO models of advanced gallbladder cancer were established, and the consistency of biological characteristics between them and primary patient samples was confirmed using pathological analysis and RNA-sequencing. Additionally, we tested the efficacy of chemotherapeutic drugs, targeted drugs, and immune checkpoint inhibitors using these two models. The results demonstrated that gemcitabine combined with cisplatin induced significant therapeutic effects. Furthermore, treatment with immune checkpoint inhibitors elicited promising responses in both the humanized mice and PDO immune models. Based on these results, gemcitabine combined with cisplatin was used for basic treatment, and immune checkpoint inhibitors were applied as a complementary intervention for gallbladder cancer. The patient responded well to treatment and exhibited a clearance of tumor foci. Our findings indicate that the combined use of PDO and PDX models can guide the clinical treatment course for gallbladder cancer patients to achieve individualized and effective treatment.

## Introduction

Gallbladder cancer is a highly aggressive biliary malignancy (1). The early symptoms of gallbladder cancer are not obvious, and thus, most patients have progressed to an advanced stage by the time of diagnosis (2, 3). Radical surgical resection of the gallbladder and regional lymph node dissection are currently the best treatments for gallbladder cancer (1); however, the recurrence rate after radical surgery remains high and is often accompanied by local or distant metastases (4). Furthermore, patients have a low response rate to postoperative chemotherapy (5). Thus, individualized therapeutic drug screening for patients with gallbladder cancer after surgery is particularly important.

Patient-derived tumor xenograft (PDX) and patient-derived tumor organoid (PDO) models serve as the best *in vivo* and *in vitro* models, respectively, for predicting clinical outcomes. Both models mimic the biological properties of the original patient tumor (PT) and maintain tumor heterogeneity (6). There is growing evidence that PDX (7–9) and PDO (10–13) models can accurately predict patient responses to anti-cancer treatment.

Currently, chemotherapy, targeted therapy, and immunotherapy are the most common drug treatments for cancer. Chemotherapeutic drugs, which suppress tumors by rapidly killing dividing cells, remain one of the main strategies for treating tumors, and our understanding of cancer pathogenesis, targeted drugs (14, 15), and immunotherapeutic drugs (16) are being developed by scientists. Targeted drugs inhibit molecular pathways that are critical for tumor growth and maintenance (17), while immunotherapy suppresses tumors by stimulating host immune responses (18). In this study, we established PDX and PDO models from a patient with advanced gallbladder cancer and conducted a multifaceted drug sensitivity trial that included chemotherapeutic agents, targeted drugs, and immune checkpoint inhibitors. The aim of this study was to guide the clinical treatment strategy for the patient by testing treatment options on the patient-derived models, with the goal of achieving individualized and effective treatment.

## Materials and methods

### Pre-operative diagnosis

Thickening, mild progressive magnetic resonance imaging (MRI) signal enhancement, and diffusion-weighted imaging hyperintensity were observed in the base and body wall of the gallbladder in one patient with gallbladder cancer. A patchy, slightly long T2 signal shadow was seen in the adjacent liver parenchyma, with mildly progressive enhancement on the enhancement scan. Slightly low signal was detected in the hepatobiliary specific phase, while high signal was detected *via* diffusion-weighted imaging, and a striped, slightly low-density shadow was observed in the adjacent right anterior lobe of the liver envelope. We observed several nodular soft tissue shadows in the upper abdomen and subperitoneum with a diameter of about 1.0 cm, high signal *via* diffusion-weighted imaging, and enlarged lymph nodes on the right side of the rectal mesentery. Pathological findings included a moderately differentiated adenocarcinoma of the gallbladder with focal findings of nerve invasion, infiltration of the entire wall of the gallbladder through to the adipose tissue with an infiltration depth of 11 mm, and tumor involvement of the liver. Cancer cells were observed in the fibrous adipose tissue of the greater omentum, and cancerous nodules were detected in the adipose tissue adjacent to the lymph nodes (pathological stage: AJCC pT3N0).

### Clinical samples

Clinical tumor samples of advanced gallbladder cancer (F210708) and human peripheral blood mononuclear cells (PBMCs) were obtained from one patient admitted to the Xijing Hospital of the Fourth Military Medical University. Informed consent was obtained from the patient, and the study was approved by the Ethics Committee of Xijing Hospital (KY20203128-1). Tumor tissue samples were used to establish PDO and PDX models, as well as for histological analysis and transcriptome sequencing.

### Laboratory animals

The animal experiments in this study were approved by the Laboratory Animals Welfare Ethics Committee of the Fourth Military Medical University (IACUC-20220259). Female NOD-*Prkdc^em26Cd52^Il2rg^em26Cd22^*/Gpt (NCG) mice (6–7 weeks old) were purchased from GemPharmatech LLC (China) and housed in the Specific Pathogen Free facility of the Laboratory Animal Center of the Air Force Medical University.

### PDO model establishment and drug screening

Patient tumor samples were excluded from necrotic areas and washed twice with phosphate-buffered saline (PBS) and were minced using a human tumor dissociation kit (Miltenyi Biotec, Germany). Single cells were dissociated using a Gentle MACS Octo Dissociator (Miltenyi Biotec) according to the manufacturer’s instructions. The dissociated tissues were washed with PBS, and the suspension was filtered through a 100 μm cell filter and centrifuged at 300 × *g* for 5 min. The supernatant was discarded and the precipitated cells were collected and resuspended in an appropriate amount of matrigel (356231, Corning, USA). Subsequently, 50 μl droplets were placed in 6-well plates, and organoid culture medium was added to cover the droplets for incubation at 37°C under 5% CO_2_. The organoid culture medium consisted of Advanced DMEM/F12 medium, 250 ng/ml Rspo-1, 100 ng/ml Wnt3a, 10 mM Y27632, 1:100 N2 supplement, 3 nM dexamethasone, 1.25 mM N-acetyl-l-cysteine, 100 ng/ml Noggin, 50 ng/ml EGF, 100 ng/ml FGF10, 10 nM gastrin, 1:50 B27 supplement, 10 mM nicotinamide, 5 μM A8301, 10 μM forskolin, 5 μg/ml prostaglandin E2, 1:500 Primocin, 1% penicillin/streptomycin, 1% Glutamax, and 1% HEPES.

The organoid cells were plated at 5×10^3^ cells/well, and the culture medium was discarded 24 h after cell treatment. Each drug was diluted in organoid culture medium at different dilution ratios (**Table S1**) and applied to the PDO culture. The CellTiter-Glo 3D kit (Promega, USA) was used to detect cell viability. Maximal doses were capped at peak plasma concentrations reported earlier in patients (19).

### Establishment of a PDO/immune cell co-culture model for drug screening

Human PBMCs were mixed with organoid cells in a 10:1 potent target ratio, resuspended in 40% matrigel, and mixed for 48 h in accordance with the density of organoid cells (5×10^3^ cells/well) for plate culture. The original medium was discarded and replaced with fresh medium containing nivolumab (**Table S1**). After 72 h, the status of the PDOs was observed microscopically, and organoid activity was detected using the CellTiter-Glo 3D kit after removal of co-cultured PBMCs using the Human Lymphocyte Isolate kit (**TBD Science, China)**.

### PDX model establishment and drug screening

Patient-derived tumor samples were removed from necrotic sites and washed twice with PBS. The specimens were minced and transplanted subcutaneously on the backs of NCG mice. When the tumors (P0) grew to approximately 500 mm^3^, tumor tissue was isolated under aseptic conditions and transplanted to the dorsal subcutis of new NCG mice (P1; n=30). When the P1 tumors reached 100–150 mm^3^, the animals were randomly divided into six groups with five animals each. Animals were treated with single or combined drugs (see **Table S2** for treatments and doses). Tumor volumes and animal body weight were measured every 3 days, and serum samples were collected at the end of the treatment period to measure CA19-9 levels by ELISA. When the P2 (n=10) generation tumors grew to approximately 500 mm^3^, the most sensitive drug was administered to observe the inhibitory effect on larger tumors. After 3 weeks of treatment with the drug, treatment was discontinued for 100 days to observe the resulting changes in the tumor.

### Establishment of a PDX model of the human immune system for drug screening

P0 generation tumors were transplanted subcutaneously into NCG mice, and 1×10^7^ PBMCs were injected into the tail vein 20 days later. After 14 days, PBMCs were isolated from blood collected *via* the tail vein. Human CD45^+^ cells were detected using flow cytometry; the mice (n=10) containing 5-10% human CD45+ cells were included in the study and randomly divided into two groups, and drugs were administered according to the schedule in **Table S2**. Tumor volume was measured three times a week. At the end of the experiment, tumors were collected for immunofluorescence staining of tumor-infiltrating human CD45^+^ and CD8^+^ cells. Additionally, PBMCs were isolated from the blood, and human CD45^+^ and CD8^+^ cells were detected using flow cytometry.

### Flow cytometric examination

Whole blood was collected from PDX mice that received the human PBMC transplant. The cells were separated using a human lymphocyte isolation solution and processed for split red blood cells. The cells were incubated with CD45^+^ (304006; BioLegend) and CD8^+^ (12-0088-42; Invitrogen) antibodies according to the manufacturer’s instructions, and flow cytometry (Attune NxT; Thermo Fisher) analysis was performed.

### Staining and histopathology

Tumor tissues and organoids were fixed in 4% paraformaldehyde (Servicebio, China) at room temperature for 24 h. Paraffin-embedded samples were cut into 5-μm sections and used for hematoxylin & eosin, immunohistology, and immunofluorescence staining. The samples were stained using antibodies specific for HER2 (2165S, Cell signaling, 1:400), CA19-9 (ab398, Abcam, 1:100), CEA (ab133633, Abcam, 1:3000), EGFR (ab32198, Abcam, 1:100), Ki67 (27309-1-AP, Proteintech, 1:2000), and PD-L1 (ab205921, Abcam, 1:500) for immunohistology staining, and with antibodies against CD45 (13917S, Cell signaling, 1:200) and CD8 (66868-1-Ig, Proteintech, 1:200) for immunofluorescence staining.

### Serum CA19-9 assay

Blood was collected from the mice at the end of the experiment, and serum was separated and sent to the Laboratory Department of Xijing Hospital for CA19-9 testing by ELISA.

### RNA transcriptome sequencing

Total RNA was extracted using an RNA extraction kit (Tiangen, China). A strand-specific library was constructed by NovelBIo (Shanghai, China), enriched and purified, and reverse-transcribed into cDNA. The cDNA libraries were quantified and validated after end-repair, purification, and enrichment, and then sequenced and analyzed.

### Statistical analysis

Statistical analysis was performed using SPSS version 18.0. Differences between two groups were analyzed using two-tailed unpaired t-tests. Differences between three or more conditions with one independent variable were analyzed using one-way analysis of variance (ANOVA); P values are reported as *<0.05, **<0.01, and ***<0.001. Image J software was used for immunofluorescence results, and the mean gray value was determined.

## Results

### Establishment of PDX and PDO models from patient gallbladder cancer tissue

**Figure 1:**
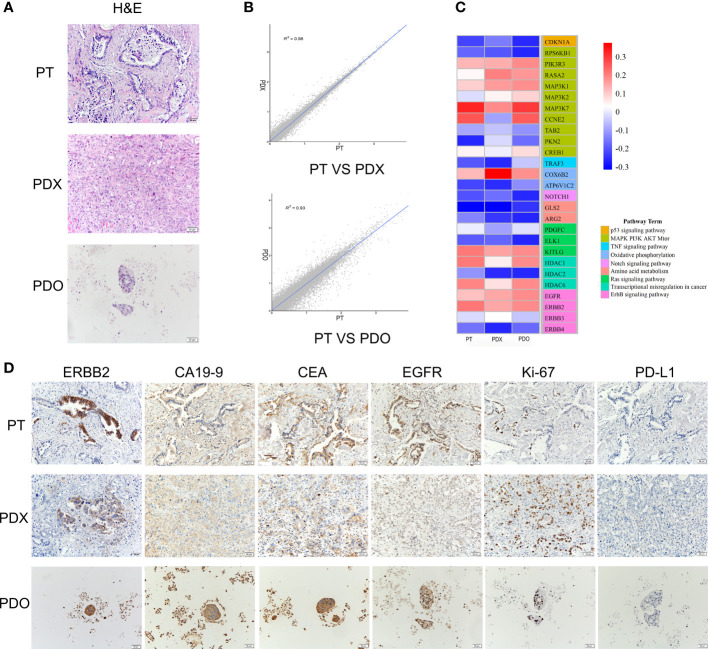
**(A)** H&E staining of the PT, PDO, and PDX samples. Scale bars, 50 μm. **(B)** Transcriptomic correlation analysis of the RNA transcriptome expression between the PDX and PT samples and between the PDO and PT samples. A total of 60,665 genes were analyzed. **(C)** A heatmap of representative genes in tumor-associated signaling pathways. RNA expression levels are indicated along a red and blue scale for high and low expression levels, respectively. **(D)** The expression of tumor markers detected using immunohistochemistry in PT, PDX, and PDO samples. Scale bar, 50 μm. H&E, hematoxylin & eosin staining; PDO, patient-derived tumor organoid; PDX, patient-derived tumor xenograft; PT, patient tumor.

Both the PDX and PDO models derived from a patient with gallbladder cancer were successfully established. The histological features of both the PDX-derived tumor tissues and the PDO were identical to those of the original PT (**Figure 1A**). Using RNA-sequencing, we detected a high correlation between the transcriptomes of tissue samples from the PDX or PDO models and primary PT samples (PDX vs. PT = 0.98; PDO vs. PT = 0.93), indicating that the models were transcriptionally representative of the original tumor (**Figure 1B**). To further confirm that tumor-associated hotspot genes from the PT were consistently expressed in the PDX and PDO models, the expression levels of genes representing several different signaling pathways were assessed. As shown in the heatmap in **Figure 1C**, the PDX, PDO, and PT samples all exhibited very similar expression patterns of genes in the p53 signaling pathway, mitogen-activated protein kinase (MAPK)-phosphatidylinositol-3-kinase (PI3K)-protein kinase B (AKT)-mammalian target of rapamycin (mTOR) signaling pathway, TNF signaling pathway, oxidative phosphorylation pathway, Notch signaling pathway, Ras pathway, and ErbB pathway. Next, we examined the expression levels of gallbladder cancer-related markers (ERBB2, CA19-9, CEA, EGFR, Ki67, PD-L1) using immunohistochemistry; all markers were detected in the PDX, PDO, and PT samples with the exception of PD-L1, which is a negative prognosticator for gallbladder cancer (**Figure 1D**). These data demonstrate that the PDX and PDO models maintained the histopathological, transcriptomic, and protein expression characteristics of the primary gallbladder tumor.

### Drug screening using the PDO model

**Figure 2:**
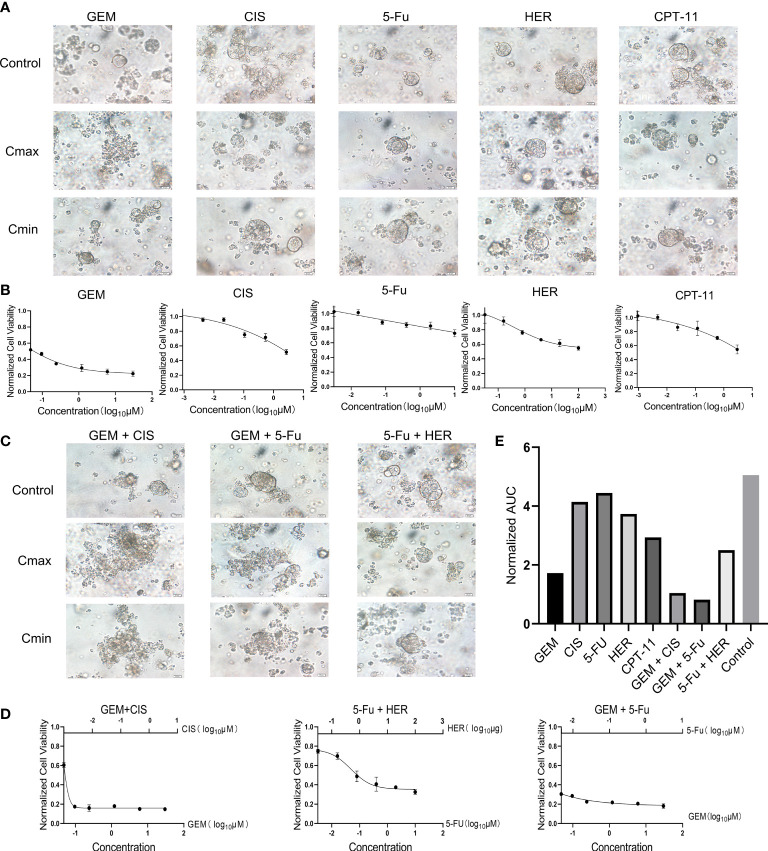
**(A)** Bright-field microscopy images of PDOs showing the results of treatment with five drugs (GEM, CIS, 5-Fu, HER, CPT-11), including controls and maximum (Cmax) and minimum concentration (Cmin). Scale bar, 20 μm. **(B)** Cell viability of PDOs after 24 h of treatment with different concentrations of each drug. **(C)** Bright-field microscopy images of PDOs showing the results of treatment with three combinations of drugs (GEM+CIS, GEM+5-Fu, 5-Fu+HER) including controls and maximum (Cmax) and minimum concentrations (Cmin). Scale bar, 20 μm. **(D)** Cell viability of PDOs after 24 h of treatment with different concentrations of combination drug treatments. **(E)** Area under the PDO cell viability curve for single and combination drug treatment. CAP, Capecitabine; CIS, Cisplatin; GEM, Gemcitabine; Cmax, maximum concentration; Cmin, minimum concentration; CPT-11, Irinotecan; HER, Trastuzumab; 5-Fu, Fluorouracil.

Next, we used our established PDO model to screen various drugs for the treatment of gallbladder cancer. Based on clinicians’ recommendations, we selected four chemotherapeutic agents (gemcitabine [GEM], cisplatin [CIS], capecitabine [CAP], irinotecan [CPT-11]) and one targeted agent (trastuzumab [HER]) for single-agent treatment. We also performed multi-agent treatments using the following drug combinations: GEM+CIS, GEM+CAP, or CAP+HER. Importantly, because the organoids lacked the relevant enzymes to catalyze CAP to 5-fluorouracil (5-FU) *in vivo* (20), CAP was replaced with 5-FU in our PDO drug screen. After treatment with a high concentration of GEM, the PDO appeared necrotic and disintegrated; conversely, the PDO remained intact and exhibited good cellular activity after treatment with high concentrations of the other drugs (**Figure 2A**). Consistent with our microscopic results, quantification of organoid activity using the CellTiter-Glo 3D kit indicated that GEM treatment achieved stronger tumor suppression than the treatment using other test drugs (P<0.001, **Figure 2B**). Furthermore, both combination treatments involving GEM elicited stronger tumor-killing effects than GEM treatment alone, and combining higher doses of 5-FU with HER also resulted in better tumor suppression than each individual drug (**Figure 2C**); organoid activity levels were consistent with these results Furthermore, we assayed the organoid activity using the CellTiter-Glo 3D kit after treatment with the drug combinations, and the results were the same as those observed by microscopy (**Figure 2D**). There was no statistical difference between the GEM+CIS and GEM+5-FU groups (P>0.05), and both were significantly different compared to the 5-FU+HER group (P<0.05). Next, we quantified the area under the curve for all drug treatment outcomes, which can be used to assess drug responses in PDO models (12). Consistent with previous results, GEM+CIS or GEM+5-FU treatment resulted in the best tumor suppression (**Figure 2E**). We predict that these findings can be used to guide the clinical treatment strategy for patients.

### Sensitivity of immune checkpoint inhibitors in PDO/PBMC co-cultures

**Figure 3:**
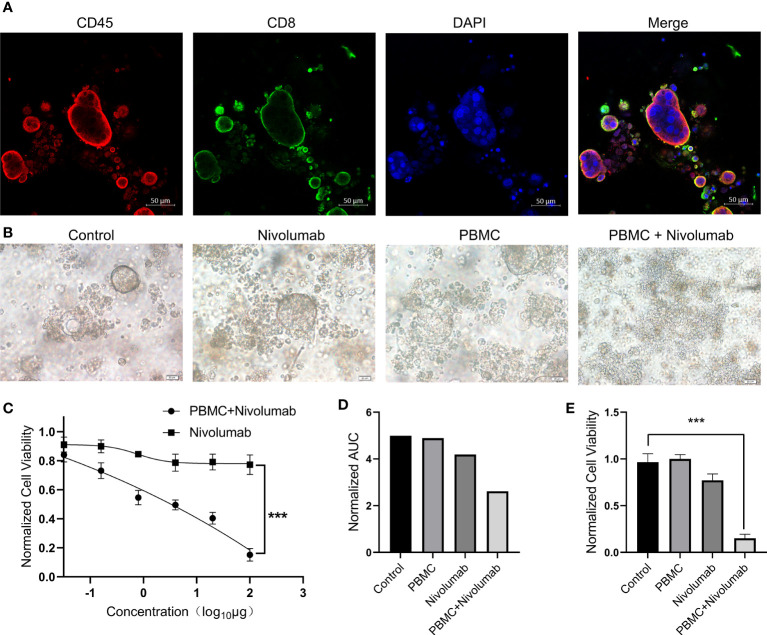
**(A)** PBMCs and PDOs were co-cultured for 48 **(h)** Immunofluorescence staining was performed for CD45^+^ cells (red), CD8^+^ cells (green), and DAPI (blue). Scale bars, 50 μm. **(B)** Bright-field microscopy images of PDOs showing the results of treatment with nivolumab. Results are shown for the control, nivolumab treatment alone, PBMC co-culture alone, and nivolumab treatment after co-culture groups. Scale bars, 20 μm. **(C)** PDO cell viability after 72 h of treatment with nivolumab. **(D)** Area under the PDO cell viability curve for each treatment group. **(E)** Cell viability in each group following nivolumab treatment at high concentrations. PDO, patient-derived tumor organoid; PMBC, peripheral blood mononuclear cell.

To investigate the efficacy of immune checkpoint inhibitors in our PDO model, we co-cultured a PDO with PBMCs. We observed that immune cells underwent fusion with the PDO, and a large number of PBMCs infiltrated the organoid (**Figure 3A**). We selected nivolumab for immunotherapy and diluted the drug according to the chart in **Table S1**. The addition of nivolumab to the co-culture resulted in PDO death (**Figure 3B**); notably, no significant tumor suppressive effect was observed after PDO treatment with nivolumab in the absence of PBMCs. Organoid activity was greatly reduced after treatment with high concentrations of nivolumab (P<0.001; **Figure 3C**). Quantification of the area under the curve indicated that nivolumab did not have a particularly prominent tumor suppressive effect on the PDO in co-culture (**Figure 3D**); however, our analysis of organoid tumor activity in co-culture indicated a highly significant reduction in PDO cell viability (P<0.001) after treatment with a high concentration of nivolumab compared to that in the control (**Figure 3E**). Thus, from these data we conclude that nivolumab had a strong tumor suppressive effect on organoids co-cultured with immune cells.

### Drug screening using the PDX model

**Figure 4:**
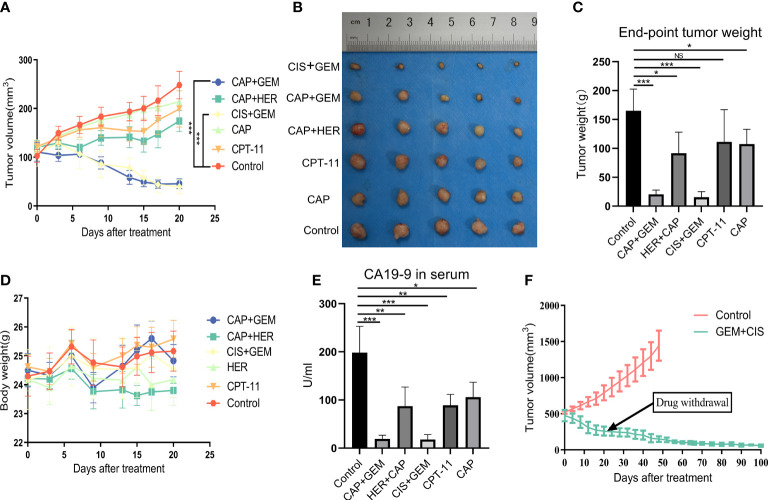
**(A)** Changes in tumor volume in each group. **(B)** Photographs of tumors in each group at the treatment endpoint. **(C)** Tumor weight in each group at the treatment endpoint. **(D)** Changes in mouse body weight in each group during treatment. **(E)** Serum CA19-9 levels in each group of mice at the treatment endpoint. **(F)** Long-term effects after the treatment of larger tumors and discontinuation of treatment. CAP, capecitabine; CIS, cisplatin; GEM, gemcitabine; CPT-11, irinotecan; HER, trastuzumab.

Tumor tissue from the PDX model was transplanted subcutaneously into the backs of 30 NCG mice (P1 generation tumors), and when the tumors had grown to approximately 100–150 mm^3^, the animals were randomly divided into six treatment groups for drug screening: control, CPT-11, CAP, GEM+CIS, CAP+HER, and GEM+CAP (**Table S2**). Significant tumor suppression was observed in the GEM+CIS and GEM+CAP groups (P<0.001 for both groups compared to that in the control; **Figure 4A**). The CAP+HER group was significantly different compared to the control group (P<0.05). The two single-agent treatments, CAP and CPT-11, were not significantly different than the control (P>0.05). Overall, these results are consistent with the results of our PDO drug screening. For all treatments, the weight of the mice remained stable (**Figure 4D**), indicating that the administered doses were within safe limits and that tumor regression was not due to general drug toxicity. At the end of the experiment, we collected the tumors and found that the change in tumor weight was consistent with the change in tumor volume (**Figures 4B, C**). Given the high expression of CA19-9 in the serum of patients with gallbladder cancer (**Figure S1B**) and in tumors (**Figure S1C**), we assessed CA19-9 levels in the serum of the mice and found that CA19-9 expression levels reflected the degree of tumor suppression, suggesting that CA19-9 expression may be indicative of tumor treatment efficacy in the PDX model (**Figure 4E**). Histopathological analysis revealed that almost no tumor cells remained in the GEM+CIS group, indicating high efficacy of the combination of GEM and CIS in tumor treatment (**Figure S1A**). In order to explore the inhibitory effect of GEM+CIS treatment on large tumors and the growth of tumors after discontinuation of treatment, we transplanted P1 tumors subcutaneously on the backs of NCG mice (P2 generation). When the tumors grew to approximately 500 mm^3^, we randomly divided the mice (n=12) into two groups. GEM+CIS treatment was discontinued after 3 weeks of treatment, and dynamic monitoring was performed for up to 100 days. The tumor volume decreased rapidly after treatment, and the tumor did not recur after discontinuation (**Figure 4F**). Overall, the trends observed in the PDX model were consistent with those in the PDO model. CA19-9 expression varied with tumor volume, indicating that CA19-9 expression may be indicative of the patient’s treatment response. These results demonstrate that GEM+CIS treatment results in long-lasting suppression of large tumors and predict that patients may have a good prognosis after the discontinuation of GEM+CIS treatment.

### Drug screening in humanized immuno-oncology models

**Figure 5:**
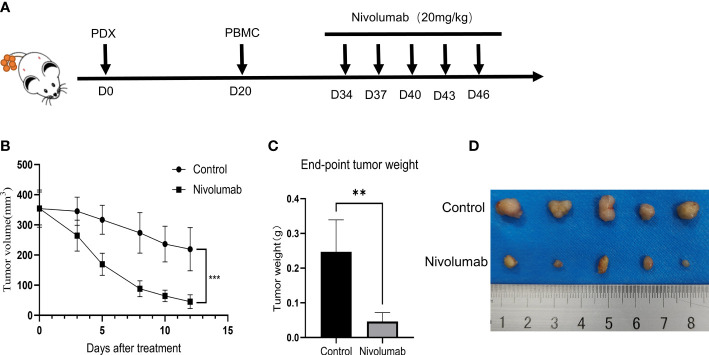
**(A)** Preparation of humanized tumor and immune system mice and drug delivery. **(B)** Change in tumor volume during treatment. **(C)** Tumor weight at the treatment endpoint. **(D)** Photographs of tumors at the end of the treatment.

**Figure 6:**
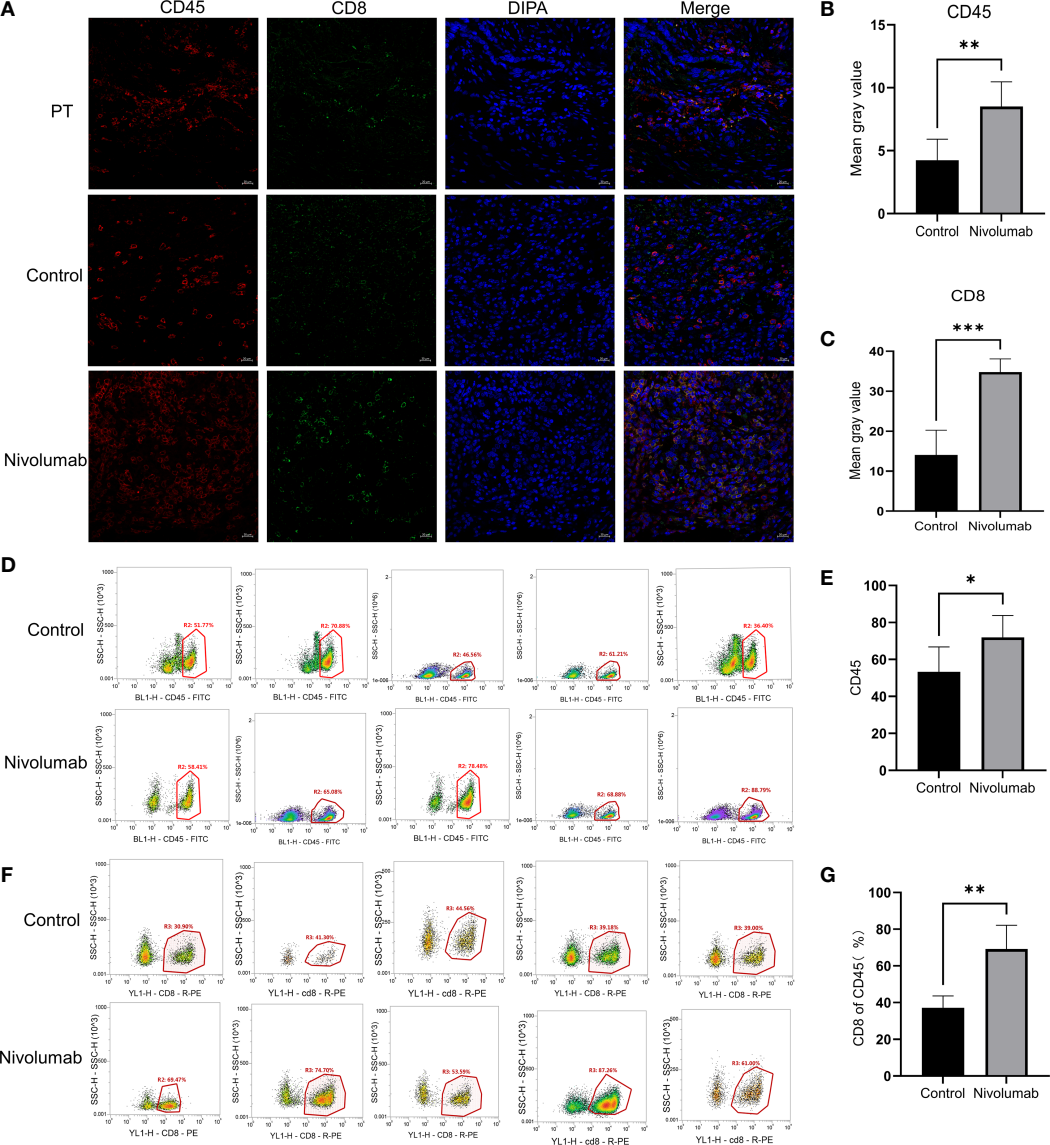
**(A)** Immunofluorescence staining of tumor sections from the PT, control, and nivolumab groups was performed for CD45^+^ cells (red), CD8^+^ cells (green), and DAPI (blue). Scale bars, 20 μm. **(B)** Quantification of CD45^+^ immunofluorescence grayscale values in the control and nivolumab groups. **(C)** Quantification of CD8^+^ immunofluorescence grayscale values in the control and nivolumab groups. **(D)** The proportion of CD45^+^ cells in PBMCs from the blood of mice in the control and nivolumab groups detected using flow cytometry. **(E)** Statistical results of the proportion of CD45^+^ cells. **(F)** Detection of the CD8^+^ ratio in CD45^+^ cells in the control and nivolumab groups using flow cytometry. **(G)** Statistics on CD8^+^ ratio in CD45^+^ cells. PT, patient tumor.

To further validate the tumor suppressive effect of the immune checkpoint inhibitor nivolumab *in vivo*, we injected human PBMCs (1 × 10^7^) into NCG mice with P1 tumors to establish a humanized immuno-oncology model (**Figure 5A**). Due to differences in the level of immune reconstitution, we included mice in which 5–10% of PBMCs comprised human CD45^+^ T cells (n=10), as determined using flow cytometry, and randomly divided them into two groups for nivolumab treatment (control and nivolumab groups). Two weeks after treatment was terminated, the tumor volume and weight in the nivolumab group were significantly less than those in the control group (P< 0.001 and P<0.01, respectively). Imaging analysis also revealed significant differences between the two groups (**Figures 5B–D**). Immunofluorescence analysis showed that the ratio of CD45^+^ to PBMC and CD8^+^ T cells to CD45^+^ was significantly higher in the nivolumab-treated group than in the control group (P<0.001 and P<0.01, respectively; **Figures 6A–C**), which further demonstrates the efficacy of immunotherapy. Meanwhile, higher proportions of CD45^+^ (P<0.05; **Figures 6D,E**) and CD8^+^ (P<0.01; **Figures 6F, G**) cells were detected in the PBMCs of mice after nivolumab treatment. These results suggest that nivolumab treatment causes immune cell death in tumors after immune reconstitution by mimicking PD-1 expression and promoting effector T cell differentiation.

### Guiding individualized clinical treatment strategies

Based on the PDO and PDX models, we found that GEM+CIS, GEM+CAP, and nivolumab treatments all achieved desired therapeutic outcomes, providing guidance for the treatment of the patient. The patient was administered GEM combined with CIS as the base treatment and the immune checkpoint inhibitor nivolumab as a complementary intervention. One year after surgery, CA19-9 returned to healthy levels (**Figure S1B**). MRI results showed a reduction of fluid in the gallbladder area and a reduction in the size of the retroperitoneal and larger lymph nodes by approximately 0.5 cm. The patient’s overall condition improved, and the prognosis was good.

## Discussion

Gallbladder cancer is the most common biliary tract tumor (21), but is commonly diagnosed at advanced stages due to the lack of obvious disease symptoms and specific markers (22). Adjuvant therapy after cholecystectomy is not routine, as most regimens have a low response rate (23); therefore, individualized therapeutic drug screening for gallbladder cancer patients after surgery is especially important. Both the PDX and the PDO models preserve the biological characteristics and heterogeneity of the original tumor and display unique advantages for individualized therapy and predicting clinical outcomes (24, 25). Unfortunately, it often takes a long time to establish a PDX model (26), and patients with a rapidly progressive disease rarely benefit from this model. While establishing a PDO model requires less time, these models cannot be used to test all drugs because they lack functional vascular, metabolic, and nervous systems (27, 28). In this study, we adopted a strategy to use PDX and PDO models together, specifically the PDO model for initial rapid drug screening and the PDX model for *in vivo* drug efficacy validation, making these two models complementary.

Both the PDX and the PDO models were used to evaluate the treatment efficacy of chemotherapeutic agents, targeted agents, and immune checkpoint inhibitors. Ultimately, we found that GEM combined with CIS exhibited the best tumor suppressive effect, while immune checkpoint inhibitors also achieved good tumor suppression. Notably, chemotherapy can stimulate anti-cancer immunity and enhance the effect of immunotherapy by stimulating cancer cells to release immunostimulatory factors or by mediating off-target effects on immune cell populations (29). We continuously observed the growth and recurrence of tumors after 3 weeks of treatment with GEM+CIS. Even 79 days after the discontinuation of GEM+CIS treatment, we did not observe tumor recurrence; these data predicted that the patient may have a good prognosis after the discontinuation of GEM+CIS treatment. Therefore, we recommended the patient for GEM+CIS-based therapy with the immune checkpoint inhibitor nivolumab as a complementary intervention, which resulted in an improvement in the patient’s condition. Because drug screening for the patients with tumor has time limit requirements, drug screening for too long will miss the best period of treatment, so we relied on clinicians’ suggestions of the candidate drugs to test. These suggestions were made based on patient-specific pathology; for instance, the patient had an ERBB2 mutation, so the ERBB2-targeting drug HER was selected. It is possible that other targeted drugs may elicit tumor suppressive effects as well.

In the process of tumor drug screening, both chemotherapeutic drugs and targeted drugs act directly on PDO models (30); therefore, the choice of drug concentration is crucial. Blindly using the IC_50_ or higher drug concentrations will likely produce clinically irrelevant results. In designing our PDO drug test, we referred to the recommendations of the drug instructions and The peak concentration in the pharmacokinetics is set as the highest concentration for our drug test (19); in this way, we were able to align our drug test results with the actual needs of the patient.

CAP, a fluoropyrimidine carbamate, is widely absorbed. CAP is converted to 5-FU *via* a multiorgan, three-step enzymatic pathway, and malignant tissues have higher concentrations of thymidine phosphorylase, which is involved in drug conversion, so 5-FU increases enrichment at tumor sites, and CAP exhibits better tumor suppressive activity in multiple PDX models (20). However, because of the lack of an effective metabolic pathway in the PDO model, we switched from CAP to 5-FU for drug testing, which exemplifies a drawback in the PDO drug screening procedure.

Tumor immunotherapy is a novel therapeutic strategy that has shown good therapeutic efficacy in many individual patients. PD-L1 expression is an initial screening marker for tumor patients receiving immunotherapy. However, some reports have demonstrated that the expression of PD-L1 is not fully indicative of the effect of immunotherapy (31, 32). Indeed, some patients with low expression of PD-L1 still achieve good therapeutic results following PD-L1 antibody treatment. Therefore, although PD-L1 was absent in the gallbladder carcinoma tumor in this study, we included PD-L1 targeted drugs in our drug screen as an alternative treatment scheme, which achieved good therapeutic results.

Humanized immuno-oncology models recapitulate a partially functional humanized immune system and are the best preclinical immunotherapy models currently available, as they allow for the prediction of patient responses to immunotherapy (33–35). Mice with a partially functional humanized immune system constructed using PBMCs can rapidly produce human T cells and thus have been widely used in tumor immunotherapy research. A number of CD45^+^ and CD8^+^ T cells were detected in the tumor tissues of the patient, indicating immune cell infiltration into the tumor; this provided rationale for immunotherapy (36, 37). In our humanized immuno-oncology model, we observed a reduction in tumor size concurrent with increasing levels of immune reconstitution in the absence of drug intervention. This may be due to the strong antigenic properties of PDX grafts and the killing effect of mature T cells in the PDX model. However, after treatment with nivolumab, the tumor volume decreased rapidly, demonstrating the inhibitory activity of nivolumab on this tumor. Similarly, the results of immune cell analysis confirmed that levels of both CD45^+^ and CD8^+^ T cells were significantly higher after nivolumab intervention. Consistent with the data from previous studies (31, 32), these results provide further evidence that PD-L1 expression is not a direct indicator of the tumor suppressive activity of immune checkpoint inhibitors, as nivolumab sharply suppressed tumor activity despite the absence of PD-L1 in the tumor.

In conclusion, both PDX and PDO models derived from one patient with advanced gallbladder cancer were successfully established. Treatment with GEM combined with CIS had significant therapeutic effects on both models, and immune checkpoint inhibitors responded well in the PDO/immune model and humanized mice. This strategy for drug selection ultimately displayed a good curative effect on the patient. Our findings indicate that the combination of PDO and PDX models can guide the clinical treatment strategy for cancer patients to achieve effective individualized treatments.

## Data availability statement

The original contributions presented in the study are included in the article/**Supplementary Material**. Further inquiries can be directed to the corresponding authors.

## Ethics statement

The study was approved by the Ethics Committee of Xijing Hospital (KY20203128-1). The patients/participants provided their written informed consent to participate in this study. The animal experiment in this study was approved by the Laboratory Animals Welfare Ethics Committee of the Fourth Military Medical University (IACUC-20220259).

## Author contributions

DT, JA, and CS: conceptualization. MG, WH, HM, HL, and YZ: conducted experiments. XG and CS: wrote the manuscript. CZ and CS: revised the manuscript. All authors gave final approval and agreed to be accountable for all aspects of the study.
