# Supplementary material for: Screening of an individualized treatment strategy for an advanced gallbladder cancer using patient-derived tumor xenograft and organoid models
Source: Front Oncol. 2022 Dec 14;12:1043479. doi: 10.3389/fonc.2022.1043479 (PMC9795198; doi:10.3389/fonc.2022.1043479)
Supplement: Supplementary file 1 [file DataSheet_1.docx]

# Supplementary Material

**Table S1. Drug concentrations tested.**

| **Groups** | **Drug Concentrations** | | | | | |
| --- | --- | --- | --- | --- | --- | --- |
| **GEM** | 30 μM | 6 μM | 1.2 μM | 240 nM | 48 nM | 9.6 nM |
| **CPT-11** | 2.95 μM | 590 nM | 118 nM | 23.6 nM | 4.72 nM | 0.944 nM |
| **CIS** | 2.67 μM | 534 nM | 106.8 nM | 21.36 nM | 4.272 nM | 0.8544 nM |
| **5-Fu** | 10 μM | 2 μM | 400 nM | 80 nM | 16 nM | 3.2 nM |
| **HER** | 100 μg/ml | 20 μg/ml | 4 μg/ml | 0.8 μg/ml | 0.16 μg/ml | 32 ng/ml |
| **Nivolumab** | 100 μg/ml | 20 μg/ml | 4 μg/ml | 0.8 μg/ml | 0.16 μg/ml | 32 ng/ml |
| **GEM+** | 30 μM+ | 6 μM+ | 1.2 μM+ | 240 nM+ | 48 nM+ | 9.6 nM+ |
| **CIS** | 2.67 μM | 534 nM | 106.8 nM | 21.36 nM | 4.272 nM | 0.8544 nM |
| **GEM+** | 30 μM+ | 6 μM+ | 1.2 μM+ | 240 nM+ | 48 nM+ | 9.6 nM+ |
| **5-Fu** | 10 μM | 2 μM | 400 nM | 80 nM | 16 nM | 3.2 nM |
| **5-Fu+** | 10 μM+ | 2 μM+ | 400 nM+ | 80 nM+ | 16 nM+ | 3.2 nM+ |
| **HER** | 100 μg/ml | 20 μg/ml | 4 μg/ml | 0.8 μg/ml | 0.16 μg/ml | 32 ng/ml |

**Table S2. Drug solutes, routes of administration, and doses used in the PDX drug test.**

| **Groups** | **Vehicle** | **Route of Adm** | **Dose** |
| --- | --- | --- | --- |
| **CPT-11** | 0.9% NaCl | IP injection | 60 mg/kg dose, once a week |
| **CIS** | 0.9% NaCl | IP injection | 3 mg/kg dose, twice a week |
| **GEM** | 0.9% NaCl | IP injection | 30 mg/kg dose, twice a week |
| **CAP** | 0.5% CMC | PO injection | 200 mg/kg dose, once a day |
| **HER** | 0.9% NaCl | IP injection | 5 mg/kg dose, every 3 days |
| **Nivolumab** | 0.9% NaCl | IP injection | 20 mg/kg dose, every 3 days |
| **GEM+CIS** | **same as single drug** | | |
| **GEM+CAP** |  |  |  |
| **CAP+HER** |  |  |  |


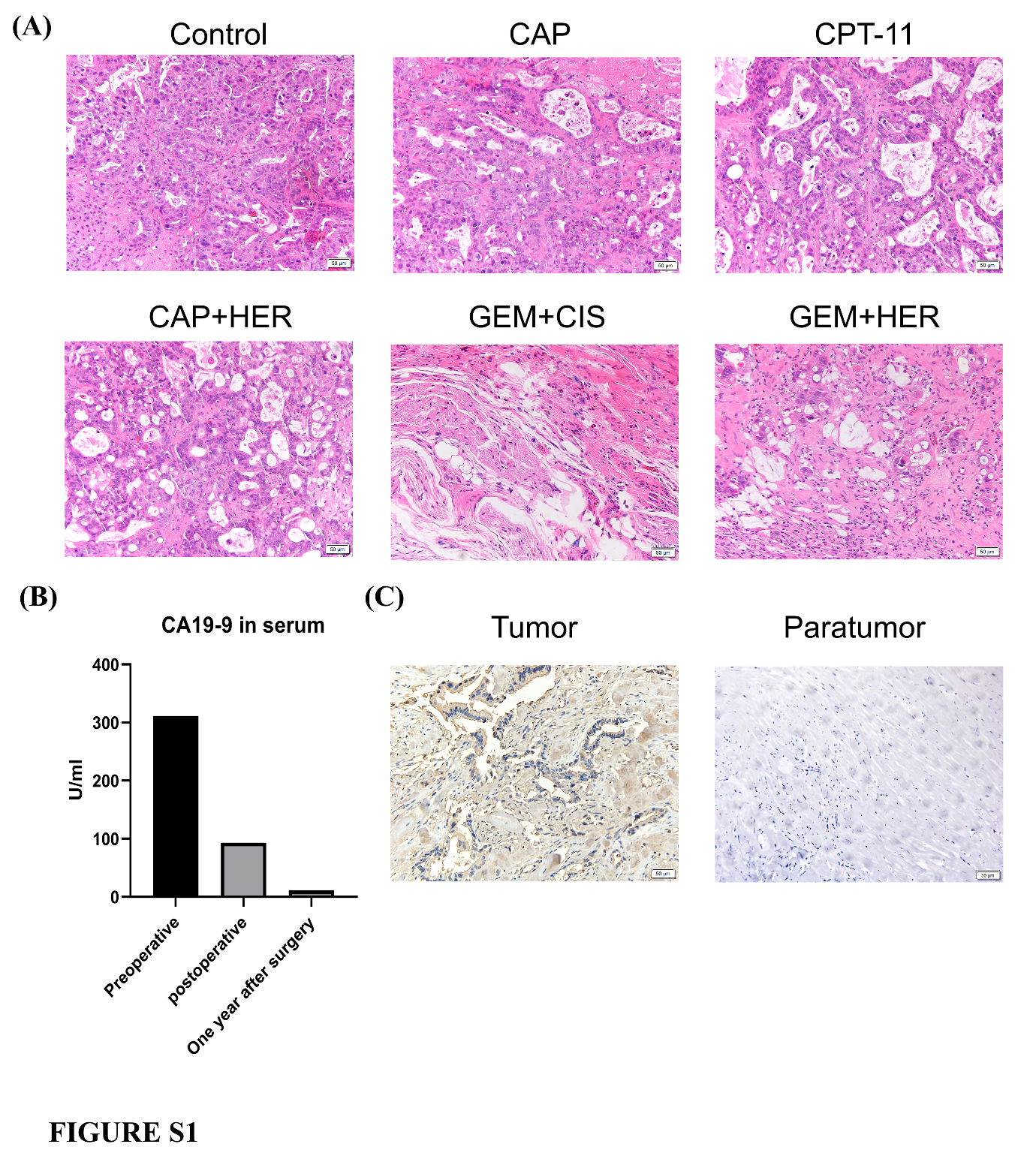


**Figure S1**

(**A**) H&E staining results of tumor sections in each group after PDX treatment. Scale bars, 50 μm. (B) Expression of CA19-9 in patients before, immediately after, and one year after surgery. (C) Expression of CA19-9 in the tumor and paratumor. Scale bars, 50 μm. CAP, capecitabine; CIS, cisplatin; GEM, gemcitabine; Cmax, maximum concentration; Cmin, minimum concentration; CPT-11, irinotecan; HER, trastuzumab; H&E, hematoxylin & eosin staining; 5-Fu, 5-fluorouracil; PDO, patient-derived tumor organoid; PDX, patient-derived tumor xenograft.
